# Reduced functional brain connectivity prior to and after disease onset in Huntington's disease^☆^^☆☆^

**DOI:** 10.1016/j.nicl.2013.03.001

**Published:** 2013-03-14

**Authors:** Eve M. Dumas, Simon J.A. van den Bogaard, Ellen P. Hart, Roelof P. Soeter, Mark A. van Buchem, Jeroen van der Grond, Serge A.R.B. Rombouts, Raymund A.C. Roos, on behalf of the TRACK-HD investigator group

**Affiliations:** a Department of Neurology, Leiden University Medical Centre, Leiden, The Netherlands; b Department of Radiology, Leiden University Medical Centre, Leiden, The Netherlands; c Institute of Psychology, Leiden University, The Netherlands; d Leiden Institute for Brain and Cognition (LIBC), The Netherlands

**Keywords:** Huntington's disease, Resting state fMRI, Premanifest gene carriers, Functional connectivity

## Abstract

**Background:**

Huntington's disease (HD) is characterised by both regional and generalised neuronal cell loss in the brain. Investigating functional brain connectivity patterns in rest in HD has the potential to broaden the understanding of brain functionality in relation to disease progression. This study aims to establish whether brain connectivity during rest is different in premanifest and manifest HD as compared to controls.

**Methods:**

At the Leiden University Medical Centre study site of the TRACK-HD study, 20 early HD patients (disease stages 1 and 2), 28 premanifest gene carriers and 28 healthy controls underwent 3 T MRI scanning. Standard and high-resolution T1-weighted images and a resting state fMRI scan were acquired. Using FSL, group differences in resting state connectivity were examined for eight networks of interest using a dual regression method. With a voxelwise correction for localised atrophy, group differences in functional connectivity were examined.

**Results:**

Brain connectivity of the left middle frontal and pre-central gyrus, and right post central gyrus with the medial visual network was reduced in premanifest and manifest HD as compared to controls (0.05 > p > 0.0001). In manifest HD connectivity of numerous widespread brain regions with the default mode network and the executive control network were reduced (0.05 > p > 0.0001).

**Discussion:**

Brain regions that show reduced intrinsic functional connectivity are present in premanifest gene carriers and to a much larger extent in manifest HD patients. These differences are present even when the potential influence of atrophy is taken into account. Resting state fMRI could potentially be used for early disease detection in the premanifest phase of HD and for monitoring of disease modifying compounds.

## Highlights

• We applied resting state fMRI in premanifest and manifest Huntington's disease.
• Reduced functional brain connectivity was present in premanifest HD gene carriers.
• Large regions demonstrate reduced functional brain connectivity in manifest HD.
• Medial visual, default mode and executive control networks were affected.

## 1 Introduction

Huntington's disease (HD) is an autosomal dominant neurodegenerative disease characterised by progressive motor-, behavioural- and cognitive-dysfunction. The expansion of the HTT gene on chromosome 4 is eventually responsible for neuronal loss and dysfunction throughout the brain (Ross and Tabrizi, 2011; van den Bogaard et al., 2012). Previous studies have demonstrated that atrophy of both the deep grey matter structures and of the cortex are apparent in patients with HD, and also to a lesser degree in HD gene carriers prior to disease onset (Aylward et al., 2000; Rosas et al., 2002; Tabrizi et al., 2011; van den Bogaard et al., 2010). These premanifest gene carriers, who do not show symptoms of the disease but are certain of eventual disease onset, have also been found to show reduced integrity of white matter(Dumas et al., 2012; Reading et al., 2005; Rosas et al., 2006). In patients with HD, both extensive white matter integrity loss and atrophy of white matter have been shown (Beglinger et al., 2005; Dumas et al., 2012; Weaver et al., 2009).

Clinical assessments in multiple functional domains have extensively objectified the impairments reported by patients and their companions (Caine et al., 1978; Ho et al., 2003; Kirkwood et al., 2001; Snowden et al., 2001). Also in premanifest gene carriers numerous tests of functioning have shown diminished performance (Brandt et al., 2002; Paulsen et al., 2008; Solomon et al., 2008). In an effort to bridge the gap between the observed clinical deteriorations and structural brain deficits, a number of studies have applied clinical assessments whilst observation of brain activity was performed using functional magnetic resonance imaging (fMRI).

Four task-based fMRI studies in manifest HD demonstrated a fairly homogenous profile, with reductions in brain activation in numerous cortical and sub-cortical brain regions (Clark et al., 2002; Kim et al., 2004; Thiruvady et al., 2007; Wolf et al., 2008b). However the results from the limited number of task-based fMRI studies in premanifest HD report a more heterogeneous pattern. Increased activation in several brain regions was found in premanifest gene carriers far from expected disease onset, and reduced activation was reported in premanifest gene carriers close to expected disease onset (Paulsen et al., 2004). These task-based fMRI studies all challenged the brain during the MRI scanning yielding assessment and performance dependent results. An alternative approach, which is currently gaining an interest is to examine the brain connectivity patterns without taxation (Seibert et al., 2012; Wolf et al., 2011). There are major differences in the knowledge base that can be gained from task-based fMRI as opposed to resting state fMRI. RS fMRI examines the functional interactions between brain regions, whereas task-based fMRI targets regional differences activated by a task (Biswal et al., 2010). A further major difference is that task-based fMRI has the added confounder of interference of disease state on task performance. This is not the case with resting state fMRI as no active input is required.

Brain function depends on large-scale brain interactions (Mesulam, 1998). Functional brain connectivity patterns can be examined at rest with fMRI and this approach is recognized as an important step towards understanding functional brain networks (Biswal et al., 2010). Hence, recent reports have incorporated resting state (RS) fMRI to examine the brain during both normal ageing and disease (Damoiseaux et al., 2008; Hafkemeijer et al., 2011; Veer et al., 2010; Zhang and Raichle, 2010). Currently the earliest robustly detectable brain changes in HD are atrophy of subcortical grey matter structures (Paulsen et al., 2008; Tabrizi et al., 2009). Given that cell loss presents as the result of a pathologic cascade it is plausible that functional brain changes may occur prior to cell loss. In carriers of the APOE-4 gene, alterations in intrinsic functional connectivity have been observed even in the absence of changes in brain structure (Filippini et al., 2009). Only a limited number of studies have investigated this in premanifest HD (Seibert et al., 2012; Wolf et al., 2011), whereby one study only examined premanifest HD (Seibert et al., 2012) and one study used a perfusion based MRI approach instead of a RS-fMRI technique (Wolf et al., 2011). Functional brain changes may also occur in HD, either prior to, or as a result of brain atrophy. RS fMRI has the potential to give insight into potential functional changes. This exploratory study aims to establish whether functional brain connectivity at rest is altered in both premanifest HD gene carriers and early manifest patients.

## 2 Material and methods

### 2.1 Participants

At the Leiden University Medical Centre study site of the TRACK-HD study, subjects participating in the longitudinal TRACK-HD study underwent MRI scanning including fMRI during the baseline visit. Of the 90 participants included, 11 did not undergo the additional fMRI scan due to time constraints. Furthermore after quality control of the fMRI data, both visually and by means of the scan analysis reports generated during post-processing of the MRI data, three manifest HD participants were excluded from the analysis because of excessive motion (maximum motion during scan > 4 mm (Jenkinson et al., 2011)). In total 20 disease stage 1 and 2 HD patients, 28 premanifest gene carriers and 28 healthy controls were included in the fMRI analysis (Table 1).

Inclusion criteria for HD patients included a positive genetic test for the HTT gene with 40 or more CAG repeats; the presence of motor disturbances defined as more than five points on the Unified Huntington's Disease Rating Scale—total motor score (UHDRS-TMS), and a Total Functional Capacity score (TFC) greater than or equal to seven points, thereby only including patients in the earliest two disease stages (Shoulson and Fahn, 1979). Inclusion criteria for premanifest gene carriers consisted of a positive genetic test with 40 or more CAG repeats, and the absence of motor disturbances with five or less points on the UHDRS-TMS. Finally, a burden of pathology score ((CAG repeat length − 35.5) × age) greater than 250 (Penney et al., 1997) was required. Age- and gender-matched gene-negative relatives of HD gene carriers and unaffected spouses were included as healthy controls. Exclusion criteria for all participants included previous significant head injury, any other neurological or major psychiatric disorder, or unwillingness to undergo MRI scanning. Medical history taking, an interview based assessment and questionnaires were used to ascertain that no major psychiatric disorder could be classified at the time of inclusion and scanning. Consequently the use of neuroleptic medications or antidepressants was sparse and considered to be of no influence. The study was approved by the Medical Ethical Committee of the Leiden University Medical Centre. All participants gave written informed consent. For full details of study parameters see Tabrizi et al. (2009).

During further medical history taking, handedness was recorded by means of the Edinburgh Inventory 2nd version (Oldfield, 1971). For early HD patients, the rater's estimate of disease onset was determined, based on the rater's observations, reports by the patients and information from companions or relatives. With this information the current disease duration was calculated. For premanifest gene carriers the estimated number of years until disease onset was calculated based on their current age and CAG repeat length, by means of the formula developed by Langbehn et al. (2004).

### 2.2 MRI protocol

MRI acquisition was performed on a 3 T whole body scanner (Philips Achieva, Healthcare, Best, The Netherlands) with an eight channel receive array head coil. An anatomical T_1_-weighted scan was acquired using an ultrafast gradient echo 3D acquisition sequence with the following imaging parameters: repetition time (TR) = 7.7 ms, echo time (TE) = 3.5 ms, field-of-view = 24 × 24 × 16.4 cm, and matrix size 224 × 224, with a duration of 9 min. For post-processing registration purposes, a high resolution T1-weighted scan, with the following parameters was collected; repetition time (TR) = 2200 ms, echo time (TE) = 30 ms, field-of-view = 220 × 220 × 168 mm, flip angle = 80°, and matrix size = 112 × 109mm, with a duration of 46 s. A RS fMRI scan with the following parameters was also obtained: repetition time (TR) = 2200 ms, echo time (TE) = 30 ms, field-of-view = 220 × 220 × 10.4 cm, resolution = 1.96 × 1.96 × 2, no slice gap, flip angle = 80°, and matrix size 80 × 79, with a duration of 7.5 min. To reduce unnecessary sensory input that could influence the results, participants were not allowed to listen to music during the RS fMRI scan, and to ensure a wakeful disposition participants were asked to keep their eyes open with normal background light.

### 2.3 Pre-processing for RS fMRI analysis

Pre-processing of the RS fMRI data using the standard procedure was carried out using FSL 4.1.8 (Smith et al., 2004). The following steps were preformed: head motion correction (Jenkinson et al., 2002), brain extraction (Smith, 2002), and spatial smoothing using a Gaussian kernel of 5 mm full width at half maximum (FWHM). All volumes were normalised based on mean intensity and high-pass temporal filtering (Gaussian-weighted least-squares straight line fitting, FWHM = 100 s). The middle (reference scan) of each individual's RS fMRI time series was affine registered to MNI152 standard space (Montreal Neurological Institute, Montreal, QC, Canada): initially, it was registered to the high resolution T1-weighted scan. This high resolution T1-weighted scan was subsequently registered to the anatomical T1-weighted scan. Finally, the anatomical scan was registered to MNI152 standard space. By first registering the functional data to the high resolution scan and then to the anatomical T1-weighted scan allows for better registration of the data. These three registration matrices were combined to obtain a matrix for transforming fMRI data from native space to standard space, using interpolation to 2 × 2 × 2 mm voxels. Subjective visual quality control was performed on all scans to ensure correct registration.

### 2.4 Statistical analyses

Statistical analysis of group demographics and the movement parameters during scanning derived from the MRI preprocessing were compared using SPSS (version 17, SPSS, USA). Where appropriate either Analysis of Variance or Chi-squared tests were applied.

**Fig. 1:**
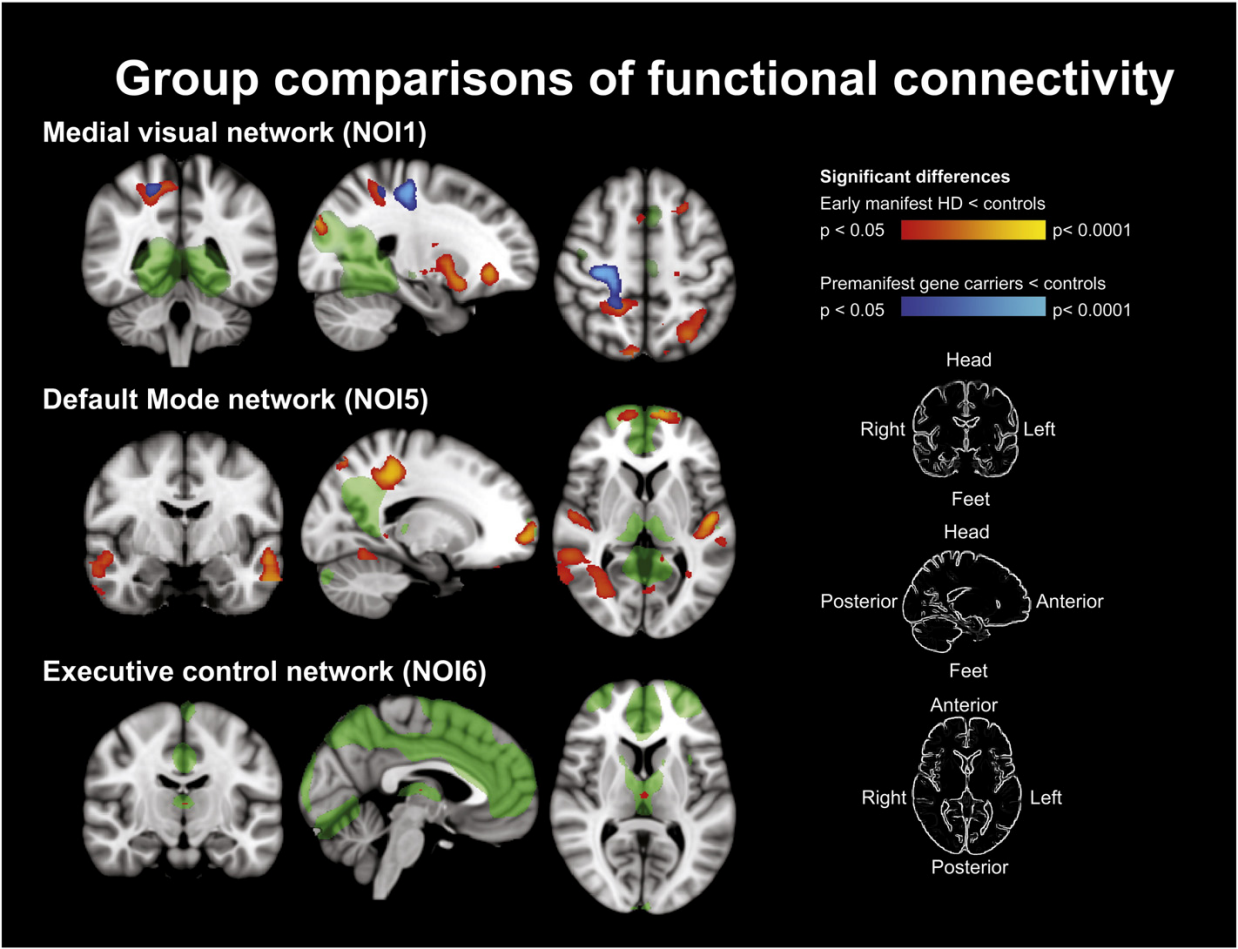
Group comparisons of functional brain connectivity shown in three orientations. Green areas show the voxels encompassing the network of interest (NOI) with which the connectivity decreases are present. Blue–light blue areas show the areas of reduced connectivity with the NOI between premanifest gene carriers and controls, red–yellow areas show the areas of reduced connectivity with the NOI between early manifest HD and controls. Some areas of blue and red overlap is present, here the functional connectivity is reduced in both premanifest and manifest HD.

Resting state connectivity was examined using a dual regression method (Filippini et al., 2009; Khalili-Mahani et al., 2011; Zuo et al., 2010). In doing so the similarity of the haemodynamic response patterns (fMRI signal) for each brain voxel was compared to the fMRI signal in eight pre-defined, well-established, networks of interest (NOIs) (Beckmann et al., 2005). These networks encompass over 80% of the entire brain volume. The eight NOIs represent spatial template maps corresponding to medial visual (NOI1), lateral visual (NOI2), auditory (NOI3), sensorimotor (NOI4), the default mode network (NOI5), executive control (NOI6), visual–spatial memory (NOI7), and working memory (NOI8) networks. See Fig. 1 for visual display of NOI1, NOI5 and NOI6.

First, a spatial regression was applied: The eight NOIs and a single CSF voxel (left ventricle horn) were used as spatial regressors in a general linear model (GLM) to obtain the nine corresponding dynamic patterns of fMRI signal fluctuations in each network from each individual's RS fMRI scan. Next, these nine time series, together with six motion correction parameters derived during preprocessing (three translations and three rotations) were used as temporal regressors in a second (temporal) GLM. For each voxel, the z-score corresponding to each of these 15 temporal regressors was obtained. A GLM was applied, resulting in spatial z-score maps for each individual's RS fMRI scan, for each NOI. This dual-regression method thereby generated eight z-scores maps reflecting the connectivity strength of each voxel in the brain to each of the eight NOIs. A voxel with a high z-score demonstrated a highly similar pattern of fMRI fluctuation to the voxels in the NOI.

The z-score maps were constructed to compare the groups. The group statistical analysis was performed to determine which brain regions showed statistically significant differences in connectivity to any of the NOIs between groups by applying three independent sample t-tests. Non-parametric permutation based statistical inference was used with 5000 repeated permutations per NOI for the comparisons; controls vs premanifest, controls vs manifest HD and premanifest vs manifest HD. Correction for multiple comparisons per NOI was applied using threshold free cluster enhancement based correction whereby all results under the threshold of p < 0.05 were considered statistically significant (Smith and Nichols, 2009). Due to the exploratory nature of the study a further correction of the comparison of the eight networks was not applied in order to prevent inflation of type II errors. From the resulting areas of statistical differences the z-scores were extracted per individual per network, and the average group values of the z-scores are displayed in Table 3. The analysis of the network connectivity was performed with a voxel-wise correction for localised grey matter to rule-out any potentially confounding impact of local structural loss on brain connectivity, as described by Oakes et al. (2007). This correction method has also been applied in Alzheimer's disease (Damoiseaux et al., 2012) and ALS (Cosottini et al., 2012). In short, per individual the anatomical T1-weighted scans were processed to provide grey matter voxel-based probability maps, which were included as a voxel-wise covariate in the mixed effects model group analysis. To subsequently determine the spatial location of differences in the voxel-wise covariate these were statistically analysed by applying three independent sample t-tests. Non-parametric permutation based statistical inference was used with 5000 repeated permutations group for the comparisons; controls vs premanifest, controls vs manifest HD and premanifest vs manifest HD. As in the previous analysis; correction for multiple comparisons was applied using threshold free cluster enhancement based correction whereby all results under the threshold of p < 0.05 were considered statistically significant (Smith and Nichols, 2009). Overall the entire procedure provided spatial information per NOI of brain regions demonstrating different connectivity patterns between the study groups.

## 3 Results

The groups were not statistically different in terms of age, gender, handedness and education level. Early HD patients had significantly higher UHDRS motor scores, CAG repeat lengths and lower Total Functional Capacity scores than premanifest gene carriers and/or healthy controls. Statistical differences were also found in the amount of movement during scanning between groups, whereby the early manifest group displayed higher amounts of maximum, absolute and relative movement as compared to premanifest gene carriers and controls. Premanifest gene carriers did not display more movement than controls (Table 2).

Premanifest gene carriers and early manifest HD displayed an overlap in a region of reduced connectivity with NOI1 (medial visual network) in the left frontal lobe and the right parietal lobe, as compared to controls (0.05 > p > 0.0001). The area in the left frontal lobe comprised the grey matter near the pre-central and middle-frontal gyri. The area in the parietal lobe was localised in the post-central gyrus, and showed the highest levels of statistical difference (Fig. 1). Premanifest gene carriers only, also displayed reduced connectivity bi-laterally of the cingulate gyrus with NOI1 compared to the controls. This area of reduced connectivity was not found in the early HD group. The manifest HD group demonstrated additional areas of reduced connectivity with NOI1 that were not observed in the premanifest gene carrier group. These areas were located bi-laterally within the superior occipital lobe, within a large field in the deep grey matter, including the putamen, globus pallidus, thalamus, and bi-laterally in the cortex of the frontal orbital region. The deep grey matter areas displayed the highest levels of statistical significance (Fig. 1).

The connectivity of the left parietal lobe, the pre-frontal cortex in both hemispheres, and regions of grey and white matter in the both temporal lobes with NOI5 (the default mode network) was reduced in early HD only as compared to controls (0.05 > p > 0.0001). The areas showing reductions bilaterally where also the areas with displaying the highest levels of statistical difference (Fig. 1).

Connectivity of a small region in the thalamus and the left supramarginal gyrus with NOI6 (executive control network) was reduced in manifest HD as compared to controls (p < 0.05) (Fig. 1).

No differences in connectivity were found with any of the NOIs when premanifest gene carriers and manifest HD were directly compared. No differences between any of the study groups were found in the connectivity with the other NOIs.

**Fig. 2:**
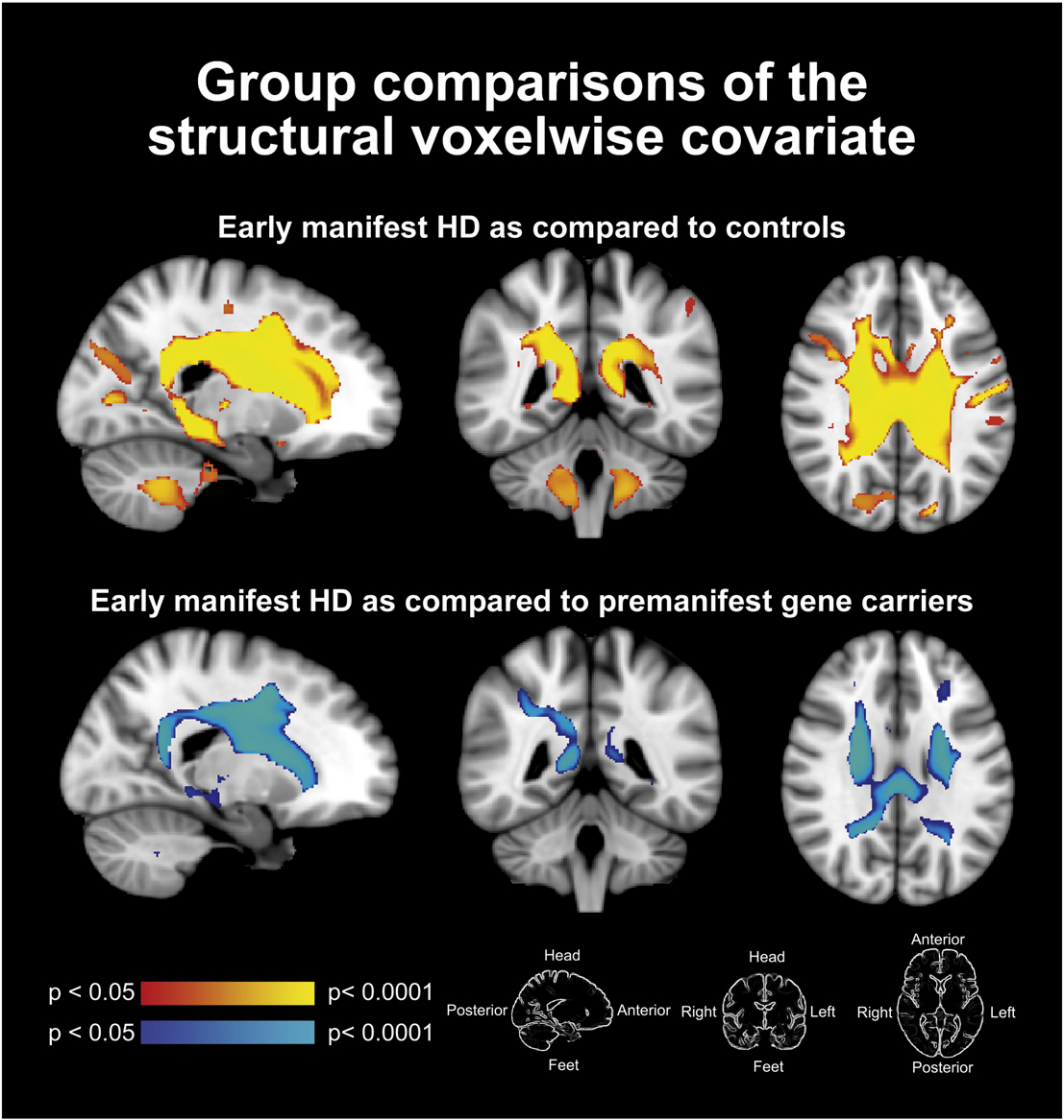
Structural voxelwise covariate differences between groups. Red–yellow areas show the areas of differences in grey matter probability between early manifest HD and controls. Blue–light blue areas of structural differences between early manifest HD and premanifest gene carriers. No areas of differences in the voxel-based probability maps where found between premanifest gene carriers and controls.

Group differences were found in the voxel-wise covariate whereby the early manifest HD group demonstrated reduced values of grey matter probability in many deeper lying brain regions as compared to controls, and as compared to premanifest gene carriers. The spatial location of these reductions is shown in Fig. 2.

## 4 Discussion

Reductions in intrinsic functional connectivity are apparent in both premanifest gene carriers and patients with early HD. The earliest areas to show a reduction in connectivity are regions within the left frontal and right parietal and bilateral visual cortices. These areas also demonstrated reduced connectivity in the early manifest group. Further connectivity reductions were also apparent in many other brain regions in early HD such as subcortical grey matter and the occipital lobes. These observed differences to healthy controls are not explained by brain atrophy.

In premanifest gene carriers our findings show reduced connectivity of NOI1 (medial visual network) with the left frontal, right parietal and bilateral cingulate gyrus during rest. The only known other report of RS fMRI in HD is a methodological report describing the stability and suitability of RS fMRI over a one year follow up period in premanifest gene carriers and healthy controls. This report by Seibert et al. (2012) reported no differences between premanifest HD and healthy controls, which contradict our findings. However, there are several methodological differences that could explain this discrepancy. Two main differences exist; first, a surface based analysis in native space rather than volume atlas transformation to MNI152 space was applied by Seibert et al. (2012). Their approach entails reduction of data points from thousands of voxels to approximately 80 regions. This is beneficial for reducing the need for many multiple comparisons, however can ‘average out’ important results. The seed regions defined by Seibert et al. (2012) are anatomically defined, however anatomical definition does not necessarily produce functional regions. In our study we choose to use established functional networks as a starting point instead of anatomical regions. We acknowledge the advantage of not registering to a volume atlas such as the MNI152 space excludes the potential for small local registration errors. Using a native space parcellation can be more readily visually inspected but can also contain small quality control rater dependent errors. The surfaced based approach and the subsequent statistical analysis applied by Seibert et al. (2012) do not include a regressor for the presence of atrophy. The second main difference is that Seibert et al. (2012) used two seed regions, again likely to be different to functional regions, namely the putamen and the isthmus cingulate. By using the cingulate as a seed, the default mode network is examined and by using the putamen one of the most affected regions in HD is addressed. In every anatomical study the putamen comes out as the strongest potential biomarker (Van den Bogaard et al., 2012). However, this approach did not result in significant differences, which seems counterintuitive as the putamen is severely affected in the premanifest stage. This severe anatomical difference does not yield functional differences which is puzzling. Other methodological differences between these two studies are; inclusion criteria and number of participants, different scanner type.

Whilst the two papers seem very different in their approach, the results are not as different as one might suspect. The main conclusions from the study by Seibert et al. are methodological, namely the longitudinal stability of resting state, there are some findings suggestive of differences in the premanifest gene carriers' resting state fMRI parameters. Modest differences were found. A weakening of the resting state correlation between the caudate and putamen was observed in premanifest gene carriers, and furthermore subjects with this weakening correlation were closer to the predicted expected disease onset. The fact we do find significant results in the premanifest group is possibly explained by the fact we applied a network approach rather than a seed approach.

Carriers of genes resulting in neurological diseases other than HD have been found to show aberrant intrinsic functional connectivity in the absence of disease signs (Filippini et al., 2009; Whitwell et al., 2011), thus supporting the occurrence of functional brain changes prior to a disease manifestation. Results from other studies using task based fMRI in HD also support our findings (Paulsen et al., 2004; Saft et al., 2008; Wolf et al., 2007, 2008a). These studies show disrupted activation (either increased or decreased) in areas that do not form an identical spatial match to our results, but do show great similarity of involved brain areas.

Some regions with altering between-region connections in premanifest gene carriers in this study, such as the left frontal lobe, specifically in the middle frontal gyri and pre-central gyrus, have also shown locally decreased task related fMRI activation (Paulsen et al., 2004; Saft et al., 2008; Wolf et al., 2007, 2008a). Task based fMRI studies also demonstrated the implication of the post central gyrus (Saft et al., 2008), and the bilateral cingulate cortex (Paulsen et al., 2004; Saft et al., 2008). Further support for our finding is found in results using a different imaging technique that measures blood perfusion during rest. Cerebral blood flow was found to be altered in premanifest gene carriers in prefrontal brain regions (Wolf et al., 2011). Our results demonstrate that early reductions in intrinsic functional connectivity are present prior to the clinical manifestation of HD. This is an important finding as therapeutic interventions may wish to monitor the functional impact of a compound on the brain in the absence of clinical outcome measures.

In the early HD group, our findings of reduced connectivity encompass more and larger regions in the brain than of premanifest gene carriers. Some, but not all of these regions have previously been shown to show disturbed activation during task based fMRI. The disrupted activation was reported in the same brain areas with which we found reductions in connectivity to NOI1 (medial visual network); left frontal lobe (Clark et al., 2002; Georgiou-Karistianis et al., 2007), right parietal lobe (Clark et al., 2002; Georgiou-Karistianis et al., 2007), superior occipital (Clark et al., 2002) and frontal orbital (Kim et al., 2004) regions in both hemispheres, and specific subcortical structures such as the putamen (Kim et al., 2004; Saft et al., 2008). However, no previous literature describes functional involvement of the globus pallidus, or thalamus. Although structural changes have been established repeatedly(Kassubek et al., 2005; van den Bogaard et al., 2010, 2012). The brain regions demonstrating reduced connectivity with NOI5 (default mode network) were also reported to show altered activation during performance, such as with the left parietal (Georgiou-Karistianis et al., 2007), and bilateral prefrontal cortices (Saft et al., 2008; Thiruvady et al., 2007) and temporal lobes (Kim et al., 2004). The reduction of connectivity of the left supramarginal gyrus and thalami with NOI6 (executive control network) during rest, does not find support in other studies of connectivity or brain activation during task execution. Despite the different nature of RS fMRI versus task based fMRI, our current findings do seem complementary to the task-based fMRI results (van den Bogaard et al., 2012). With RS fMRI overall brain connectivity is examined that is not limited to task related brain regions, and we have demonstrated that the connectivity of multiple brain networks is affected in HD.

The brain regions demonstrating reduced connectivity as compared to healthy controls visually displayed overlap between premanifest gene carriers and early HD patients, possibly indicating progressive functional deficits. The regions demonstrating reduced connectivity generally occur throughout the brain and, especially in manifest HD are present bilaterally.

It is unknown whether reduced connectivity patterns reflect connectivity that is limited or non-existent due to neuronal death or whether such results reflect intact but abnormally functioning neurons in HD. The results from this current study suggest that the latter may be a more accurate reflection, given that atrophy reflects (advanced) volume loss as a result of neuronal death, and that our results remain valid when taking into consideration MRI detectable regional atrophy. Therefore, it is not likely that reductions in functional connectivity can be explained solely by neuronal death in HD.

The strengths of this study lay in the comprehensive and exploratory nature of the fMRI analysis. As this study was performed in a single sample of strictly selected premanifest and early manifest HD the results reflect varying stages of disease progression. Furthermore, by taking atrophy into consideration the potential influence of cell loss on connectivity results was reduced. Examination of brain networks encompassing almost the entire brain allowed for a hypothesis generating approach. Therefore the brain regions found to display reduced connectivity may be targeted in future studies of HD. The analysis method was chosen based on the standard analysis procedures that allow for replication thereby enhancing the reproducibility in light of biomarker research. The limitations of this study lay in the potential for the influence of motion artefacts due to chorea. However, every effort was made to prevent motion during scanning and furthermore strict quality control was applied to prohibit the inclusion of poor quality scans. This resulted in the exclusion of three scans from the manifest HD group. Also, the influence of excessive motion was reduced by including a strictly selected early HD group where chorea is generally limited. Having said this, the presence of reduced functional connectivity in the premanifest gene carrier group – who do not display significant movement disorder – suggests that the findings may not arise as a result of movement only. Another limitation is the unknown effect of medication on functional differences. The indication and use of medication are highly variable and therefore we did not choose to control for the influence of medication. Furthermore, the eight networks examined are not specifically validated in the HD population, however these networks have been systematically found in healthy subjects (Damoiseaux et al., 2006) as well as disease states such as Mild Cognitive Impairment and Alzheimer's disease (Binnewijzend et al., 2012). Other issues are the novelty of the technique, the novelty of the eight network approach, the analysis of each of the eight networks separately whereby the potential for type II errors is increased, the use of ‘eyes-closed’ instruction during scanning and the cross-sectional design. To further understand if connectivity patterns are indeed affected by the progressive nature of this degenerative disease, study reproduction and longitudinal follow-up is essential in all study groups. Longitudinal follow-up using RS fMRI has the advantage over task-based fMRI that it is easier to standardise for cross-site, cross-cultural studies. Correlation of RS fMRI changes with changes in clinical measures will be addressed in a future longitudinal study. In the multi-site follow-up of the TRACK-HD study the evaluation of RS fMRI as a biomarker for HD is ongoing.

## 5 Conclusion

We have demonstrated that in the absence of processes that put demand on the brain, the HD brain functions differently. These differences are apparent even when the potential influence of atrophy is taken into account. We have shown that these functional differences are present not only after disease manifestation but also in the preceding ‘premanifest’ phase. Functional connectivity measures could potentially be used for early disease detection and for monitoring of disease modifying compounds.

## Figures and Tables

**Table 1:** Group characteristics of the study groups.

N	Healthy controls	Premanifest gene carriers	Early HD patients
	28	28	20
Gender M/F	13/15	11/17	5/15
Age (years) mean (SD)	48.5 (8.5)	43.21 (8.2)	46.5 (10.6)
Education level Median (range)	(2–5)	(2–5)	(1–5)
Handedness—right Number (% of group)	25 (89%)	24 (85%)	17 (85%)
CAG repeat length Mean (SD)	n/a	42.5 (2.5)	44.1 (2.6)^b^
Total functional capacity Mean (SD)	12.9 (1.9)	12.6 (0.8)	10.2 (1.9)^a^^b^
UHDRS—motor Mean (SD)	2.5 (2.5)	2.4 (1.4)	20.3 (11.0)^a^^b^
Expected disease onset (years) mean (SD)	n/a	11.6 (4.4)	n/a
Disease duration (years) mean (SD)	n/a	n/a	6.8 (7.4)

N = number of participants, SD = Standard deviation, n/a = not applicable, UHDRS—motor = Unified Huntington's Disease Rating Scale—total motor score.

a Significantly different to controls at p < 0.05.

b Significantly different to premanifest gene carriers at p < 0.05.

**Table 2:** Movement parameters of participants during scanning.

Movement parameter (mm)	Healthy controls		Premanifest gene carriers		Early HD patients	
	Mean	SD	Mean	SD	Mean	SD
Absolute	0.36	0.25	0.29	0.19	0.59⁎	0.46
Relative	0.10	0.05	0.09	0.05	0.24⁎	0.23
Maximum	1.15	0.96	0.74	0.47	2.51⁎	2.32

SD = Standard deviation.

⁎ Significant differences to controls and premanifest gene carriers. p < 0.05.

**Table 3:** Z-scores in the regions that demonstrate differences between groups.

Network	Mask	Healthy controls		Premanifest gene carriers		Early HD patients	
		Mean	SD	Mean	SD	Mean	SD
NOI1—visual	HD vs control	1.87	0.68	1.65	0.65	1.45	0.42
	PMGC vs control	1.68	1.04	0.31⁎	0.77	1.00⁎⁎	0.53
NOI5—default	HD vs control	4.08	1.11	3.40	1.32	2.19⁎⁎	1.06
NOI6—executive	HD vs control	3.61	2.04	3.14	1.87	0.84⁎⁎	1.32

SD = Standard deviation.

⁎ Significantly different from controls.

⁎⁎ Significantly different from controls and premanifest gene carriers p < 0.05.
